# Factors Associated with Moral Disapproval of Same-Sex Sexual Behavior in Denmark: Baseline Findings from the Project SEXUS Cohort Study

**DOI:** 10.1007/s10508-025-03211-5

**Published:** 2025-07-09

**Authors:** Josefine Bernhard Andresen, Christian Graugaard, Mikael Andersson, Morten Frisch

**Affiliations:** 1 Project SEXUS Group, Department of Epidemiology Research, Statens Serum Institut, 2300 Copenhagen S, Denmark; 2 Center for Sexology Research, Department of Clinical Medicine, Aalborg University, Aalborg, Denmark

**Keywords:** Mental health, Homophobia, Prejudice, Sexual orientation, Sexual attitudes, Religion

## Abstract

Homophobia is considered a central driver of minority stress, mental health problems and suicidality among non-heterosexual people. We used baseline questionnaire data from 31,808 participants aged 15–89 years in the nationally representative Danish cohort study *Project SEXUS* to identify factors associated with moral disapproval of same-sex sexual behavior, an assumed proxy for homophobia. Respondents who answered that they did “not at all” or only “to a low extent” find same-sex sexual activity morally acceptable were operationally defined as expressing moral disapproval of same-sex sexual behavior, whereas respondents who considered same-sex sexual activity morally acceptable “to a high extent” or “to a very high extent” were considered not to hold such moral reservations. Demographically weighted prevalence estimates of sociodemographic, health-related, sexual, moral, and religious characteristics among individuals with and without moral reservations concerning same-sex sexual behavior were calculated and compared in polytomous logistic regression analyses. Overall, 21.7% (women: 14.4%; men: 29.2%) expressed moral disapproval of same-sex sexual behavior. Upon adjustment for potential confounding variables, such moral reservations were significantly positively associated with late sexual debut, low lifetime number of sex partners, lacking sex education in school, missing communication with parents about sex and, among men, a poor or non-existent sex life within the last year. Individuals expressing moral disapproval of same-sex sexual behavior also expressed significantly more restrictive moral attitudes toward other sexual matters. Religiousness was strongly positively associated with moral reservations concerning same-sex sexual behavior, most notably among followers of Islam, Jehovah’s Witnesses, Danish Inner Mission, or a composite category of minor Christian denominations. In conclusion, even in a sexually liberal country such as Denmark, which decriminalized homosexuality in 1933 and legalized registered same-sex partnerships in 1989, moral disapproval of same-sex sexual behavior remains widespread. To reduce the negative mental health ramifications of homophobia and ensure the health and rights of all citizens, school-based sex education should stimulate social inclusion and destigmatization of sexual minorities, and religious communities should be encouraged to counter homophobia within and outside their circles.

**Supplementary Information:**

The online version contains supplementary material available at 10.1007/s10508-025-03211-5.

## Introduction

The term homophobia covers feelings, convictions, or expressions of negative or outright hostile attitudes toward homosexuality and homosexuals (Grey et al., 2013; Obeid et al., 2020). While homophobic attitudes do not necessarily translate into hostile, discriminatory, or violent behaviors, studies have indeed linked homophobia to bullying activities among adolescents in various peer contexts (Hooghe & Meeusen, 2012; Poteat & Espelage, 2007). Intimidation, discrimination, and stigmatization have repeatedly been associated with depression and suicidality among sexual minority individuals, and homophobic attitudes are widely considered a central driver of “minority stress” among gay people (Birkett et al., 2009; Meyer, 2003; Rimes et al., 2019). Moreover, homophobia may go beyond negative feelings toward homosexuality and homosexuals and more broadly encompass disapproval of anyone departing from heteronormativity, i.e., all individuals who do not adhere to socially defined heterosexual norms (Obeid et al., 2020). Interestingly, a review of all random school shootings in the USA during 1982–2001 revealed that most such episodes were the culmination of homophobic teasing and bullying against the shooters, although none of them actually identified as homosexuals (Kimmel & Mahler, 2003). In sum, homophobia may have strong negative consequences for the health, well-being, and self-worth of its targets, and far beyond, and is thus a public health concern.

A number of studies, many of which were based on non-representative samples of adolescents and young adults, have sought to characterize the dynamics and underlying drivers of homophobia drawing on various theoretical frameworks. High levels of social dominance orientation (i.e., the desire that one’s own group dominate and be superior to all other groups) and authoritarianism have been found to correlate positively with homophobic attitudes, and individuals with homophobic attitudes have been found to adhere more strongly than others to stereotypical gender-role concepts (e.g., MacInnis & Hodson, 2015; Whitley & Ægisdóttir, 2000). Also, gender differences, with men being consistently more likely than women to hold homophobic attitudes, have been observed (e.g., Hooghe & Meeusen, 2012; Kimmel & Mahler, 2003; Lingiardi et al., 2016), and a higher prevalence of homophobia has been reported among individuals with strong religious beliefs (Chaux & Léon, 2016; Whitley, 2009). Further, in a study measuring pupillary responses following exposure to sexual images, the pupil size in men with high levels of homophobia increased significantly less upon the presentation of sexual images than that of men low in homophobia, suggesting a lower overall sexual interest among homophobic individuals (Cheval et al., 2016). Theoretically, therefore, homophobia might reflect concerns about sexuality in general rather than homosexuality in particular.

Most previous homophobia research originates from the USA, while the topic remains largely uninvestigated in a Scandinavian context. With the world’s first nationwide legalization of same-sex registered partnerships in 1989 (the forerunner of same-sex marriage, which was legalized in 2012) and the gradual expansion of rights and protective legislation for sexual minority persons, homosexual individuals have become increasingly equalized with heterosexuals in Denmark. Nonetheless, large proportions of sexual minorities in this country still report bullying, discrimination, harassment, and assaults on the basis of their sexual identity, and they remain at higher risk of mental health problems such as anxiety, self-harm, and suicidality when compared with heterosexuals (Andresen et al., 2022a; Erlangsen et al., 2020; Frisch et al., 2019).

On this background, we aimed to estimate the extent to which moral disapproval of same-sex sexual behavior is present in Denmark and to identify key sociodemographic, health-related, sexual, moral, and religious factors associated with such moral reservations utilizing data from a large and nationally representative study. We used relevant baseline questionnaire data available in the nationwide Project SEXUS cohort study, using moral disapproval of same-sex sexual behavior as our proxy measure of homophobia.

## Method

### Participants

This study utilized questionnaire data from Project SEXUS, a cohort study of 15–89-year-old individuals in Denmark whose focus is on sexuality and the interplay between sexual and general health. Specifically, a probability-based sample of 62,675 individuals completed an online questionnaire at baseline in 2017–2018, resulting in an AAPOR response rate 1 of 34.6% (American Association for Public Opinion Research, 2016). The questionnaire included more than 600 items in total (Frisch, 2022). However, each participant was only presented with a median of 180 questions due to logical filters and because some questions were posed only to approximately half of the cohort, while other questions were posed to the other half (Andresen et al., 2022b; Frisch et al., 2019). Throughout the paper, as in prior articles originating from Project SEXUS (https://www.projektsexus.dk), all analyses were carried out separately for women and men with each participant’s sex determined by the officially recorded sex in the Danish Civil Registration System.

### Measures

Approximately half of the Project SEXUS participants (*N* = 31,808) were asked a series of questions about their attitudes toward various matters relating to sexuality and sexual rights, including two questions about same-sex sexual behavior. Specifically, participants were asked “To what extent do you consider it morally acceptable that two women have sex with each other?” and “To what extent do you consider it morally acceptable that two men have sex with each other?”, each with six response categories (to a very high extent, to a high extent, to some extent, to a low extent, not at all, I do not know). Participants who considered either female or male same-sex sexual behavior, or both, morally acceptable “to a low extent” or “not at all” were categorized as expressing moral disapproval of same-sex sexual behavior, whereas respondents who considered both female and male same-sex sexual behavior morally acceptable “to a very high extent” or “to a high extent” were categorized as not holding such moral reservations. The remaining respondents were categorized as having unclear attitudes toward same-sex sexual behavior, and these were treated as a separate group in the main statistical analyses. For clarity, results are presented only for respondents expressing moral disapproval of same-sex sexual behavior and those who did not hold such moral reservations.

Sociodemographic and health-related items examined included age in 10-year categories, region of residence (five categories), partner status (two categories), educational attainment (five categories), difficulties paying bills within the last year (three categories), employment status (two categories), and self-rated health (three categories). All used categorizations are shown in Table 2.

Sexual characteristics included respondents’ overall sexual experience, their other-sex sexual experience, and their same-sex sexual experience, each of which was derived from responses to one common question addressing which sexual experiences with men or women they had had since age 15 years (female sex partners only, mostly female sex partners and at least one male sex partner, approximately as many female as male sex partners, mostly male sex partners and at least one female sex partner, male sex partners only, no sexual experiences with either female or male partners, I do not know). Other examined sexual characteristics included sexual identity (heterosexual, homosexual, bisexual, asexual, I cannot be placed in the abovementioned categories, I do not know/undecided), and same-sex sexual attraction (to a very high extent, to a high extent, to some extent, to a low extent, not at all, I do not know). Heterosexually experienced individuals were asked about their age at sexual debut with a person of the other sex (< 12, 12, 13, …, 87, 88, 89 years, I do not know) and their total number of other-sex sexual partners (1, 2, 3–4, 5–9, 10–19, 20–49, 50–99, ≥ 100, I do not know).

Respondents were also asked whether they had received sex education in school (*yes, no, I do not know*), to what extent they had communicated with one or both of their parents about sex during childhood or adolescence (*to a very high extent, to a high extent, to some extent, to a low extent, not at all, I do not know*), and how they would rate their sex life within the last year (*very good, good, neither good nor bad, bad, very bad, no sex life within the last year, I do not know*). In analyses of sexual identity, we focused on heterosexual, homosexual, and bisexual respondents, and we dichotomized responses for overall sexual experience, other-sex sexual experience, same-sex sexual experience, and same-sex sexual attraction (*any, none*), categorizing respondents who reported one or more same-sex sexual experiences as having *any* same-sex sexual experience and those reporting same-sex sexual attraction “to a very high extent”, “to a high extent”, “to some extent” or “to a low extent” as having any same-sex sexual attraction, respectively. All used categorizations are shown in Table 3.

In addition to the two abovementioned questions used to identify individuals who did or did not express moral disapproval of same-sex sexual behavior, respondents were also asked about their moral attitudes and views regarding other matters relating to sexuality and sexual rights. Such moral questions covered topics such as sex outside of marriage, sex with new encounters, sex with many changing partners, sex with more than one person at the same time, porn watching, paying for sex, receiving payment for sex, and unfaithfulness, and respondents provided their views on the legality of same-sex marriage, non-therapeutic circumcision of males under 18 years, and induced abortion for unwanted pregnancy. All questions and used categorizations are shown in Table 4.

Religiousness was measured by two questions about the importance of religion in the respondents’ childhood home and in their current life, each with four response options (*very important*, *somewhat important*, *not very important*, *not at all important*). Respondents reporting that religion was *very important*, *somewhat important*, or *not very important* in either phase of their life, or both, were asked what specific religion (or religions) they had been or were currently affiliated with. Associations between specific religions and moral disapproval of same-sex sexual activity were estimated after restricting religious respondents to that vast majority who were affiliated with only one religion. All used categorizations are shown in Table 5.

### Statistical Analyses

In all analyses, we applied a weighting procedure to ensure the data’s national representativeness with regard to sex, year of birth, region of residence, marital status, cultural background, and twin status, as described in detail elsewhere (Andresen et al., 2022a, 2022b; Frisch & Andersson, 2019). Proportions of women and men who expressed moral disapproval of same-sex sexual behavior were compared using the Chi-square test. We calculated demographically weighted prevalence estimates of sociodemographic, health-related, sexual, moral, and religious characteristics among individuals who did or did not express moral disapproval of same-sex sexual behavior and performed polytomous logistic regression analyses to calculate associated odds ratios (ORs) with 95% confidence intervals (CIs), using individuals without moral reservations concerning same-sex sexual behavior as reference. Age distributions among individuals who did or did not express moral disapproval of same-sex sexual behavior were compared using Mood’s median test. In all statistical analyses underlying Tables 2, 3, 4, and 5, individuals responding “I do not know” to the sociodemographic, sexual, moral and religious questions were excluded. ORs for sociodemographic and health-related characteristics were adjusted for age in 10-year categories, and ORs for sexual, moral, and religious characteristics were adjusted for age in 10-year categories, region of residence, current partner status, educational attainment, difficulties paying bills within the last year, and self-rated health, and these adjusted ORs are referred to as aORs in the following.

We estimated the proportion of individuals whose moral concerns about sexual matters were particularly restrictive concerning same-sex sexual behavior. Operationally, this subgroup comprised (1) individuals who considered same-sex sexual activity to be “not at all” morally acceptable while consistently finding all other examined sexual matters morally acceptable “to a very large extent,” “to a large extent,” “to some extent,” “to a low extent” or “I do not know,” and (2) individuals who considered same-sex sexual activity to be morally acceptable “to a low extent” while consistently finding all other examined sexual matters morally acceptable “to a very large extent,” “to a large extent,” “to some extent” or “I do not know.” Proportions of women and men who expressed particularly strong moral disapproval of same-sex sexual behavior were compared using the chi-square test.

In a series of robustness analyses, we repeated all the analyses underlying our main findings in Tables 2, 3, 4, and 5, in which we included respondents with unclear attitudes toward same-sex sexual behavior in the group considered to express moral disapproval of such behavior using binomial logistic regression analyses. All statistical analyses were carried out using the *survey* and *svyVGAM* packages in R version 4.3.0.

## Results

Overall, 6,576 of the 31,808 respondents, corresponding to a demographically weighted proportion of 21.7% (women: 14.4%; men: 29.2%), considered sexual activity between two persons of the same sex to be morally acceptable “to a low extent” or “not at all”, thus meeting our criteria for expressing moral disapproval of same-sex sexual behavior. In contrast, 19,787 respondents, or 60.2% (women: 68.0%; men: 52.1%), expressed no such moral reservations, whereas the remaining 18.1% (women: 17.6%; men: 18.7%) had unclear attitudes. Sex differences with respect to these moral judgments of same-sex sexual behavior were highly statistically significant (χ^2^ = 1,139, df = 2, *p* < 0.001). Respondents with moral reservations concerning same-sex sexual activity comprised 0.5% (women: 0.3%; men: 0.8%) with moral disapproval of female same-sex sexual behavior only, 5.6% (women: 0.7%; men: 10.7%) with moral disapproval of male same-sex sexual behavior only, and 15.5% (women: 13.4%; men: 17.7%) with moral reservations concerning both female and male same-sex sexual behavior (Table 1).

**Table 1:** Demographically weighted proportions who did or did not express moral disapproval of same-sex sexual behavior among 31,808 15–89-year-old individuals, Project SEXUS, Denmark 2017–2018

	Moral disapproval of same-sex sexual behavior				
	No	Yes			Unclear attitudes
		Moral disapproval of female same-sex sexual behavior only	Moral disapproval of male same-sex sexual behavior only	Moral disapproval of both female and male same-sex sexual behavior	
	*n* (%)	*n* (%)	*n* (%)	*n* (%)	*n* (%)
Women	12,326 (68.0)	38 (0.3)	104 (0.7)	1901 (13.4)	2648 (17.6)
Men	7461 (52.1)	121 (0.8)	1594 (10.7)	2818 (17.7)	2797 (18.7)
Total	19,787 (60.2)	159 (0.5)	1698 (5.6)	4719 (15.5)	5445 (18.1)

Proportions of respondents who morally disapproved of same-sex sexual activity increased markedly with age and, among both women and men, the median age was considerably higher among individuals with than among those without such moral reservations (women: 65 vs. 44 years; men: 58 vs. 40 years) (*p* values < 0.001). After adjustment for age, both women and men expressing moral disapproval of same-sex sexual activity were significantly more likely than those without such moral reservations to live outside the capital region, to have 10 or fewer years of educational attainment, to report difficulties paying bills within the last year, and to rate their health as bad or very bad. Additionally, women with moral reservations concerning same-sex sexual behavior were more likely to be unemployed, and men with such reservations were more likely to be single (Table 2).

**Table 2:** Sociodemographic and health-related characteristics among 15–89-year-old individuals who did or did not express moral disapproval of same-sex sexual behavior, Project SEXUS, Denmark 2017–2018

	Women			Men		
	Moral disapproval of same-sex sexual behavior		OR [95% CI]	Moral disapproval of same-sex sexual behavior		OR [95% CI]
	No	Yes		No	Yes	
	*n* (%^a^)	*n* (%^a^)		*n* (%^a^)	*n* (%^a^)	
Total	12,326 (68.0)	2043 (14.4)		7461 (52.1)	4533 (29.2)	
*Age*						
15–24 years	2390 (84.5)	163 (6.5)	0.51 [0.42–0.63]	1135 (68.6)	257 (15.8)	0.40 [0.34–0.48]
25–34 years	2279 (83.3)	180 (7.4)	0.59 [0.49–0.72]	1281 (71.7)	285 (16.5)	0.41 [0.34–0.48]
35–44 years	2307 (81.1)	224 (8.3)	0.68 [0.56–0.82]	1459 (65.8)	418 (19.5)	0.52 [0.45–0.60]
45–54 years	2283 (73.7)	348 (11.1)	1 [Ref]	1391 (51.6)	747 (29.3)	1 [Ref]
55–64 years	1822 (66.6)	382 (13.7)	1.36 [1.16–1.60]	1203 (44.5)	875 (32.9)	1.30 [1.14–1.48]
65–74 years	983 (48.6)	453 (22.9)	3.12 [2.65–3.67]	732 (29.9)	1073 (45.4)	2.67 [2.34–3.05]
≥ 75 years	262 (28.9)	293 (36.3)	8.32 [6.74–10.28]	260 (16.8)	878 (58.4)	6.14 [5.18–7.26]
Median	44 years	65 years	*p* < 0.001	40 years	58 years	*p* < 0.001
*Region of residence*						
Capital Region of Denmark	4140 (75.4)	455 (10.9)	1 [Ref]	2766 (65.1)	844 (19.2)	1 [Ref]
Region Zealand	1622 (64.0)	317 (15.2)	1.62 [1.36–1.93]	923 (47.6)	685 (31.7)	2.11 [1.83–2.43]
Region of Southern Denmark	2423 (62.5)	503 (17.2)	1.90 [1.63–2.21]	1362 (44.0)	1166 (35.1)	2.69 [2.37–3.04]
Central Denmark Region	2762 (68.7)	464 (14.5)	1.54 [1.31–1.80]	1692 (49.9)	1208 (32.6)	2.30 [2.04–2.59]
North Denmark Region	1379 (62.3)	304 (17.6)	1.99 [1.66–2.39]	718 (43.3)	630 (33.9)	2.59 [2.24–2.99]
*Partner status*						
Spouse/Partner	9024 (68.1)	1528 (14.1)	1 [Ref]	5776 (50.6)	3637 (30.1)	1 [Ref]
Single	3233 (67.8)	506 (15.1)	0.85 [0.75–0.97]	1644 (56.5)	873 (26.5)	1.24 [1.11–1.38]
*Educational attainment*						
≤ 10 years	1354 (53.9)	465 (23.4)	3.16 [2.67–3.73]	900 (39.9)	1108 (38.8)	2.09 [1.84–2.38]
Secondary education	1446 (82.1)	113 (7.9)	1.54 [1.20–1.97]	884 (71.0)	225 (15.8)	0.78 [0.65–0.94]
Short-cycle higher education	2197 (59.8)	555 (18.0)	1.82 [1.59–2.08]	1029 (49.0)	696 (32.1)	1.17 [1.03–1.32]
Medium-cycle higher education	5130 (69.5)	775 (13.1)	1 [Ref]	2834 (48.3)	1923 (31.4)	1 [Ref]
Long-cycle higher education	2120 (83.2)	113 (6.2)	0.43 [0.34–0.54]	1768 (66.5)	512 (18.5)	0.36 [0.31–0.41]
*Difficulties paying bills within the last year*						
Not at all	10,034 (67.0)	1712 (14.9)	1 [Ref]	6425 (51.4)	3995 (29.6)	1 [Ref]
Sometimes	1828 (72.5)	266 (12.2)	1.28 [1.09–1.50]	881 (57.0)	433 (25.6)	1.11 [0.97–1.27]
Often	290 (74.8)	45 (13.0)	1.51 [1.07–2.13]	117 (54.3)	83 (36.5)	1.90 [1.39–2.60]
*Employment status*^*b*^						
Working	8445 (78.1)	946 (9.1)	1 [Ref]	5284 (59.2)	2167 (23.8)	1 [Ref]
Unemployed	2525 (75.9)	341 (11.0)	1.43 [1.24–1.65]	1133 (63.7)	391 (20.0)	0.98 [0.84–1.13]
*Self-rated health*						
Very good or good	9921 (69.5)	1524 (13.5)	1 [Ref]	6051 (53.5)	3433 (27.9)	1 [Ref]
Neither good nor bad	1735 (63.1)	349 (16.4)	1.22 [1.06–1.42]	1050 (47.7)	800 (33.0)	1.22 [1.09–1.36]
Bad or very bad	670 (61.1)	170 (20.3)	1.60 [1.31–1.95]	360 (45.6)	300 (36.7)	1.29 [1.09–1.54]

*OR* Odds ratio adjusted for age in 10-year intervals, *CI* Confidence interval

^a^Demographically weighted row percentages do not sum up to 100 because the category of respondents with unclear attitudes is not shown

^b^Analysis restricted to persons under the age of 65 years

After controlling for potential confounding by age, sociodemographic factors, and self-rated health, women with moral concerns over same-sex sexual activity were more likely than women without such concerns to never have had sex with another person (aOR = 3.87, 95% CI [2.90–5.18]), while no similar association was seen among men (aOR = 1.24 [0.95–1.61]) (Table 3). The vast majority of individuals with moral reservations concerning same-sex sexual behavior self-identified as heterosexuals, and they reported to have had any same-sex sexual experience (women: aOR = 0.34 [0.26–0.45]; men: aOR = 0.13 [0.10–0.18]) or any same-sex sexual attraction (women: aOR = 0.38 [0.32–0.45]; men: aOR = 0.15 [0.12–0.19]) significantly less often than individuals without such moral reservations.

**Table 3:** Sexual characteristics among 15–89-year-old individuals who did or did not express moral disapproval of same-sex sexual behavior, Project SEXUS, Denmark 2017–2018

	Women			Men		
	Moral disapproval of same-sex sexual behavior		aOR [95% CI]	Moral disapproval of same-sex sexual behavior		aOR [95% CI]
	No	Yes		No	Yes	
	*n* (%^a^)	*n* (%^a^)		*n* (%^a^)	*n* (%^a^)	
*Overall sexual experience*						
None	585 (66.1)	132 (18.0)	3.87 [2.90–5.18]	376 (60.6)	153 (21.8)	1.24 [0.95–1.61]
Any	11,709 (68.6)	1852 (13.9)	1 [Ref]	7072 (51.7)	4345 (29.5)	1 [Ref]
*Sexual identity*^*b*^						
Heterosexual	11,152 (69.5)	1726 (13.2)	1 [Ref]	6685 (51.8)	4211 (29.2)	1 [Ref]
Homosexual	302 (93.4)	9 (4.9)	0.36 [0.15–0.87]	473 (95.6)	17 (2.3)	0.05 [0.02–0.11]
Bisexual	463 (89.9)	23 (5.3)	0.51 [0.32–0.80]	196 (81.7)	24 (9.2)	0.18 [0.11–0.28]
*Other-sex sexual experience*						
None	672 (66.7)	138 (18.0)	3.59 [2.73–4.73]	627 (65.4)	159 (19.0)	0.86 [0.67–1.10]
Any	11,622 (68.6)	1846 (13.9)	1 [Ref]	6821 (51.3)	4339 (29.8)	1 [Ref]
*Same-sex sexual experience*						
None	10,720 (66.7)	1914 (14.9)	1 [Ref]	6488 (50.3)	4425 (30.5)	1 [Ref]
Any	1574 (87.5)	70 (4.7)	0.34 [0.26–0.45]	960 (82.9)	73 (7.0)	0.13 [0.10–0.18]
*Same-sex sexual attraction*						
None	7730 (61.6)	1766 (17.3)	1 [Ref]	5804 (47.9)	4341 (32.2)	1 [Ref]
Any	4508 (87.5)	226 (5.6)	0.38 [0.32–0.45]	1615 (83.1)	139 (7.0)	0.15 [0.12–0.19]
*Age at sexual debut with a person of the other sex*^*c*^						
< 12 years	73 (69.5)	13 (13.8)	1.17 [0.59–2.32]	48 (42.3)	39 (36.3)	1.52 [0.87–2.65]
12–14 years	1995 (81.4)	177 (7.8)	0.84 [0.69–1.01]	917 (57.8)	402 (24.1)	0.96 [0.83–1.12]
15–17 years	6544 (71.5)	888 (11.7)	1 [Ref]	3297 (54.1)	1871 (27.7)	1 [Ref]
18–20 years	2354 (60.8)	514 (17.9)	1.38 [1.19–1.59]	1785 (47.1)	1345 (32.9)	1.04 [0.94–1.16]
> 20 years	579 (48.8)	239 (27.7)	2.92 [2.34–3.64]	738 (43.2)	657 (36.8)	1.30 [1.12–1.50]
*Total number of partners of the other sex*^*c*^						
1	973 (49.7)	400 (26.1)	4.07 [3.30–5.02]	589 (43.3)	578 (36.2)	1.95 [1.66–2.31]
2	970 (55.1)	270 (22.5)	3.23 [2.60–4.01]	477 (43.2)	419 (35.9)	1.87 [1.56–2.23]
3–4	1911 (60.3)	419 (17.1)	2.37 [1.98–2.84]	1047 (46.3)	843 (33.7)	1.48 [1.30–1.68]
5–9	2988 (72.7)	379 (10.8)	1.49 [1.25–1.78]	1654 (52.8)	950 (28.0)	1.12 [1.00–1.27]
≥ 10	4413 (82.7)	314 (6.7)	1 [Ref]	2907 (57.7)	1363 (25.3)	1 [Ref]
*Rating of current sex life*						
Very good or good	6215 (72.8)	916 (12.5)	1 [Ref]	3881 (56.3)	1914 (25.9)	1 [Ref]
Neither good nor bad	2734 (69.7)	396 (12.4)	0.87 [0.75–1.01]	1871 (51.4)	1115 (28.5)	1.08 [0.97–1.20]
Bad or very bad	1471 (74.2)	189 (11.2)	0.85 [0.71–1.03]	1120 (51.8)	732 (31.0)	1.14 [1.00–1.30]
No sex life within the last year	1807 (54.6)	496 (21.0)	1.08 [0.91–1.29]	550 (37.1)	737 (41.2)	1.58 [1.33–1.86]
*Received sex education in school*						
Yes	10,711 (75.2)	1345 (10.6)	1 [Ref]	6313 (58.9)	2682 (23.8)	1 [Ref]
No	1129 (41.8)	585 (29.2)	1.61 [1.37–1.89]	917 (28.2)	1675 (48.7)	1.64 [1.44–1.87]
*Communication with parents about sex*						
To a very high or high extent	1283 (78.7)	134 (8.8)	0.90 [0.71–1.13]	373 (70.4)	88 (15.7)	0.77 [0.57–1.04]
To some extent	2906 (75.3)	359 (10.8)	1 [Ref]	1195 (63.7)	398 (20.0)	1 [Ref]
To a low extent	4217 (69.5)	637 (13.2)	0.98 [0.83–1.15]	2692 (57.7)	1239 (24.8)	1.07 [0.92–1.25]
Not at all	3830 (60.3)	881 (18.7)	1.22 [1.04–1.43]	3144 (43.6)	2761 (35.9)	1.48 [1.28–1.71]

*aOR* Odds ratio adjusted for age in 10-year intervals, region of residence, partner status, educational attainment, difficulties paying bills within the last year and self-rated health, *CI* Confidence interval

^a^Demographically weighted row percentages do not sum up to 100 because the category of respondents with unclear attitudes is not shown

^b^Sexual identity categories: *Asexual, I cannot be placed in the abovementioned categories*, and *I do not know/undecided* were not included in the analyses

^c^Among individuals with heterosexual experience

Among respondents with heterosexual experience, individuals expressing moral disapproval of same-sex sexual activity were significantly more likely to have had sexual debut at or after age 18 years (women) or after age 20 years (men), and they had consistently lower total numbers of sex partners than individuals without such moral reservations. Also, men with moral concerns over same-sex sexual behavior were more likely to report no sex life within the last year (aOR = 1.58 [1.33–1.86]) and, among those who did have a sex life, to rate their current sex life as either bad or very bad (aOR = 1.14 [1.00–1.30]). Individuals with moral concerns over same-sex sexual activity were more likely to have received no sex education in school (women: aOR = 1.61 [1.37–1.89]; men: aOR = 1.64 [1.44–1.87]), and to never have communicated with their parents about sexual matters during childhood or adolescence (women: aOR = 1.22 [1.04–1.43]; men: aOR = 1.48 [1.28–1.71]).

Moral disapproval of same-sex sexual activity was strongly positively linked to having restrictive moral attitudes toward other sexual matters (Table 4). Having sex with someone without being married to that person was considered morally unacceptable by 72.5% of female and 78.9% of male respondents with moral concerns over same-sex sexual behavior as compared with only 12.3% of female and 7.7% of male respondents without such moral concerns (women: aOR = 52.69 [41.02–67.67]; men: aOR = 23.47 [18.36–30.01]). Restrictive moral attitudes among individuals with moral reservations concerning same-sex sexual activity also pertained to other examined sexual activities, most notably so for having sex with more than one person at the same time (women: aOR = 38.88 [30.72–49.20]; men: aOR = 33.21 [28.59–38.57]) and watching porn (women: aOR = 84.50 [70.04–101.95]; men: aOR = 51.67 [39.71–67.24]). Most respondents, regardless of their views on same-sex sexual activities, considered transactional sex as morally unacceptable, but individuals with moral reservations concerning same-sex sexual activity were even less likely than those without such reservations to regard transactional sex as morally acceptable, whether considering paying for sex (women: aOR = 0.14 [0.11–0.17]; men: aOR = 0.20 [0.18–0.23]) or being paid for sex (women: aOR = 0.13 [0.11–0.16]; men: aOR = 0.15 [0.13–0.17]). Individuals with moral concerns over same-sex sexual activity were significantly more likely to believe that same-sex marriage should not remain legal (women: aOR = 61.14 [41.31–90.49]; men: aOR = 28.29 [22.13–36.17]), that it should remain legal for parents to have their sons under 18 years circumcised without a medical indication (women: aOR = 2.90 [2.24–3.76]; men: aOR = 1.74 [1.40–2.17]), and that abortion due to unwanted pregnancy before the end of gestational week 12 should not remain legal in Denmark (women: aOR = 15.10 [11.93–19.12]; men: aOR = 7.39 [5.66–9.64]). In a supplementary analysis, we restricted all analyses shown in Table 4 to respondents for whom religion had been and currently was “not at all important”, finding similar results (not shown).

**Table 4:** Moral attitudes toward other matters related to sexuality and sexual rights among 15–89-year-old individuals who did or did not express moral disapproval of same-sex sexual behavior, Project SEXUS, Denmark 2017–2018

	Women			Men		
	Moral disapproval of same-sex sexual behavior		aOR [95% CI]	Moral disapproval of same-sex sexual behavior		aOR [95% CI]
	No	Yes		No	Yes	
	*n* (%^a^)	*n* (%^a^)		*n* (%^a^)	*n* (%^a^)	
*To what extent do you consider it morally acceptable…*						
*That some people have sex without being married to each other?*						
To a very high or high extent	12,001 (78.8)	887 (7.0)	1 [Ref]	7227 (60.8)	2802 (22.0)	1 [Ref]
To some extent	149 (17.2)	299 (41.1)	18.43 [14.15–24.02]	105 (11.2)	571 (58.7)	9.65 [7.49–12.44]
To a low extent or not at all	145 (12.3)	783 (72.5)	52.69 [41.02–67.67]	108 (7.7)	1060 (78.9)	23.47 [18.36–30.01]
*That some people have sex with a person they just met?*						
To a very high or high extent	8082 (90.9)	232 (3.0)	1 [Ref]	6131 (70.7)	1419 (15.8)	1 [Ref]
To some extent	2865 (63.8)	467 (11.7)	4.32 [3.61–5.17]	982 (31.8)	1329 (39.0)	4.28 [3.82–4.79]
To a low extent or not at all	1099 (31.6)	1267 (43.7)	27.26 [22.81–32.59]	287 (13.3)	1657 (70.9)	17.26 [14.66–20.31]
*That some people have many changing sex partners?*						
To a very high or high extent	6792 (94.7)	140 (2.2)	1 [Ref]	5384 (77.8)	831 (11.7)	1 [Ref]
To some extent	3132 (71.5)	282 (7.3)	3.52 [2.81–4.42]	1375 (39.4)	1171 (30.9)	4.30 [3.82–4.84]
To a low extent or not at all	2005 (38.5)	1549 (36.2)	25.19 [20.57–30.85]	595 (17.3)	2402 (64.8)	17.66 [15.44–20.20]
*That some people have sex with more than one person at the same time?*						
To a very high or high extent	7013 (96.8)	105 (1.7)	1 [Ref]	5872 (80.3)	738 (10.0)	1 [Ref]
To some extent	2600 (72.9)	177 (5.2)	3.65 [2.79–4.77]	994 (34.8)	934 (31.2)	6.28 [5.52–7.15]
To a low extent or not at all	2027 (37.6)	1693 (36.7)	38.88 [30.72–49.20]	454 (12.5)	2680 (70.5)	33.21 [28.59–38.57]
*That some people watch porn?*						
To a very high or high extent	9993 (92.1)	251 (2.5)	1 [Ref]	7151 (69.0)	1796 (16.9)	1 [Ref]
To some extent	1653 (46.4)	465 (15.6)	10.44 [8.71–12.51]	213 (9.5)	1236 (52.6)	17.12 [14.47–20.25]
To a low extent or not at all	499 (21.0)	1233 (62.0)	84.50 [70.04–101.95]	82 (5.1)	1382 (84.7)	51.67 [39.71–67.24]
*That some people pay other persons for sex?*						
To a very high or high extent	2365 (88.3)	134 (5.4)	0.14 [0.11–0.17]	3199 (70.8)	811 (17.2)	0.20 [0.18–0.23]
To some extent	3179 (72.7)	284 (7.9)	0.29 [0.25–0.34]	2318 (51.7)	1272 (26.2)	0.42 [0.37–0.46]
To a low extent or not at all	6320 (64.2)	1543 (20.6)	1 [Ref]	1805 (38.4)	2326 (44.8)	1 [Ref]
*That some people receive payment for sex?*						
To a very high or high extent	2585 (89.3)	134 (5.1)	0.13 [0.11–0.16]	3518 (73.4)	783 (15.6)	0.15 [0.13–0.17]
To some extent	3524 (75.9)	256 (6.9)	0.23 [0.20–0.28]	2286 (51.1)	1282 (26.3)	0.36 [0.32–0.40]
To a low extent or not at all	5706 (61.3)	1575 (22.0)	1 [Ref]	1517 (34.3)	2330 (47.9)	1 [Ref]
*That some people are unfaithful to their partner?*						
To a very high or high extent	193 (50.3)	143 (37.5)	2.40 [1.74–3.32]	362 (48.0)	274 (38.0)	0.87 [0.70–1.08]
To some extent	1258 (75.8)	145 (10.9)	0.58 [0.47–0.72]	1372 (55.4)	634 (26.0)	0.65 [0.57–0.74]
To a low extent or not at all	10,589 (68.9)	1706 (14.4)	1 [Ref]	5520 (52.5)	3515 (29.8)	1 [Ref]
*Do you believe that, in Denmark, it should remain legal…*						
*For two persons of the same sex to marry each other?*						
Yes	12,230 (73.5)	1303 (9.7)	1 [Ref]	7290 (60.2)	2712 (20.7)	1 [Ref]
No	55 (9.2)	477 (78.1)	61.14 [41.31–90.49]	103 (6.7)	1287 (81.7)	28.29 [22.13–36.17]
*For parents to have their sons under 18 years of age circumcised without a medical indication?*						
Yes	529 (64.4)	146 (20.4)	2.90 [2.24–3.76]	426 (55.5)	219 (28.8)	1.74 [1.40–2.17]
No	11,009 (68.0)	1794 (14.3)	1 [Ref]	6514 (51.3)	4113 (29.8)	1 [Ref]
*To get an abortion due to unwanted pregnancy (before the end of 12th week of pregnancy)?*						
Yes	11,889 (71.8)	1515 (11.7)	1 [Ref]	7131 (55.4)	3712 (26.5)	1 [Ref]
No	197 (25.0)	355 (55.1)	15.10 [11.93–19.12]	125 (17.7)	468 (63.3)	7.39 [5.66–9.64]

*aOR* Odds ratio adjusted for age in 10-year intervals, region of residence, partner status, educational attainment, difficulties paying bills within the last year and self-rated health, *CI* Confidence interval

^a^Demographically weighted row percentages do not sum up to 100 because the category of respondents with unclear attitudes is not shown

As seen in Table 5, moral disapproval of same-sex sexual activity was strongly positively associated with religiousness in both women and men. Individuals with moral concerns over same-sex sexual behavior were considerably more likely than those without such concerns to report that religion had been “very important” in their childhood home (women: aOR = 6.64 [5.32–8.30]; men: aOR = 4.98 [3.98–6.22]), or that religion was currently “very important” to them (women: aOR = 9.31 [7.32–11.83]; men: aOR = 8.59 [6.54–11.29]). Compared with respondents to whom religion was “not at all important” in their childhood home, moral reservations concerning same-sex sexual activity were most pronounced among respondents who grew up under the influence of Islam (women: aOR = 35.91 [24.91–51.78]; men: aOR = 22.33 [13.97–35.72]), Jehovah’s Witnesses (women: aOR = 7.69 [4.06–14.56]; men: aOR = 9.88 [4.77–20.46]), or the composite category of minor Christian denominations (women: aOR = 7.82 [3.77–16.23]; men: aOR = 6.44 [3.55–11.68]). Further, compared with respondents to whom religion was “not at all important” currently, moral reservations concerning same-sex sexual activity were most pronounced among respondents who were currently affiliated with Jehovah’s Witnesses (women: aOR = 85.53 [22.10–315.79]; men: aOR = ∞), Islam (women: aOR = 53.89 [36.28–80.06]; men: aOR = 32.86 [19.56–55.21]), Danish Inner Mission (women: aOR = 11.97 [5.38–26.65]; men: aOR = 17.34 [4.48–67.16]), or minor Christian denominations (women: aOR = 19.24 [9.09–40.70]; men: aOR = 34.52 [15.09–78.96]).

**Table 5:** Religious characteristics among 15–89-year-old individuals who did or did not express moral disapproval of same-sex sexual behavior, Project SEXUS, Denmark 2017–2018

	Women			Men		
	Moral disapproval of same-sex sexual behavior		aOR [95% CI]	Moral disapproval of same-sex sexual behavior		aOR [95% CI]
	No	Yes		No	Yes	
	*n* (%^a^)	*n* (%^a^)		*n* (%^a^)	*n* (%^a^)	
*Importance of religion in childhood home*						
Not at all important	4578 (79.0)	436 (8.7)	1 [Ref]	3131 (64.1)	1067 (20.3)	1 [Ref]
Not very important	5504 (70.7)	729 (11.7)	1.16 [1.01–1.34]	3236 (51.4)	1986 (29.5)	1.34 [1.21–1.49]
Somewhat important	1697 (54.2)	474 (21.7)	2.02 [1.70–2.41]	798 (36.2)	934 (39.5)	1.99 [1.73–2.30]
Very important	446 (41.5)	368 (36.7)	6.64 [5.32–8.30]	255 (27.2)	474 (53.1)	4.98 [3.98–6.22]
*Specific religion in childhood home*^*b*^						
Not at all important	4578 (79.0)	436 (8.7)	1 [Ref]	3131 (64.1)	1067 (20.3)	1 [Ref]
Lutheran-evangelical Christianity (Danish State Church)	6405 (65.8)	1054 (14.8)	1.15 [1.00–1.32)	3698 (47.6)	2674 (31.9)	1.33 [1.20–1.47)
Catholicism	144 (60.1)	34 (17.1)	2.75 [1.76–4.29]	80 (44.0)	80 (36.8)	2.45 [1.68–3.57]
Danish Inner Mission	74 (45.4)	48 (35.9)	2.36 [1.46–3.81]	35 (21.6)	77 (53.3)	2.85 [1.69–4.81]
Independent Christian constituency	423 (60.7)	102 (17.0)	2.20 [1.67–2.89]	132 (37.3)	164 (42.4)	2.96 [2.23–3.92]
Jehovah’s Witnesses	28 (36.1)	39 (46.0)	7.69 [4.06–14.56]	16 (19.1)	50 (64.1)	9.88 [4.77–20.46]
Islam	81 (31.6)	117 (43.5)	35.91 [24.91–51.78]	33 (20.0)	97 (56.4)	22.33 [13.97–35.72]
Minor Christian denomination^c^	39 (46.7)	34 (37.1)	7.82 [3.77–16.23]	31 (31.8)	61 (61.0)	6.44 [3.55–11.68]
Other religion^d^	73 (61.3)	15 (11.9)	1.52 [0.76–3.04]	49 (50.1)	33 (29.2)	1.74 [1.00–3.02]
*Importance of religion today*						
Not at all important	4193 (80.8)	361 (8.2)	1 [Ref]	3723 (66.0)	1146 (19.0)	1 [Ref]
Not very important	5192 (70.6)	680 (11.5)	1.22 [1.05–1.42]	2675 (47.3)	1947 (31.9)	1.75 [1.58–1.94]
Somewhat important	2450 (59.2)	557 (18.1)	1.64 [1.38–1.94]	856 (36.1)	1007 (40.7)	2.45 [2.14–2.81]
Very important	351 (33.6)	406 (43.1)	9.31 [7.32–11.83]	157 (20.7)	384 (59.3)	8.59 [6.54–11.29]
*Specific religion today*^*b*^						
Not at all important	4193 (80.8)	361 (8.2)	1 [Ref]	3723 (66.0)	1146 (19.0)	1 [Ref]
Lutheran-evangelical Christianity (Danish State Church)	6589 (65.0)	1116 (15.0)	1.17 [1.01–1.35]	3046 (42.6)	2698 (35.3)	1.79 [1.62–1.98]
Catholicism	95 (52.1)	27 (19.1)	3.95 [2.42–6.46]	59 (45.8)	52 (33.1)	2.82 [1.78–4.48]
Danish Inner Mission	10 (16.2)	31 (69.4)	11.97 [5.38–26.65)	5 (7.2)	36 (75.8)	17.34 [4.48–67.16]
Independent Christian constituency	445 (56.8)	133 (19.5)	2.82 [2.17–3.66]	119 (34.0)	164 (46.0)	3.96 [2.94–5.32]
Jehovah’s Witnesses	3 (8.1)	41 (82.3)	85.53 [22.10–315.79]	0 (0.0)	46 (87.1)	∞
Islam	61 (25.8)	118 (48.4)	53.89 [36.28–80.06]	25 (16.7)	92 (60.4)	32.86 [19.56–55.21]
Minor Christian denomination^c^	25 (29.6)	44 (52.4)	19.24 [9.09–40.70]	10 (11.9)	49 (78.1)	34.52 [15.09–78.96]
Other religion^d^	234 (76.9)	26 (8.3)	0.80 [0.49–1.31]	122 (59.0)	46 (21.6)	1.29 [0.86–1.95]

*aOR* Odds ratio adjusted for age in 10-year intervals, region of residence, partner status, educational attainment, difficulties paying bills within the last year and self-rated health, *CI* Confidence interval

^a^Demographically weighted row percentages do not sum up to 100 because the category of respondents with unclear attitudes is not shown

^b^Analysis restricted to respondents with affiliation to only one religion

^c^Including Orthodox Christianity, Danish Lutheran Mission, The Pentecostal Movement and Seventh-day Adventism

^d^Including Judaism, Hinduism, Buddhism, Sikhism, Scientology, and Other religion

Of the total of 6,576 individuals (2,043 women; 4,533 men) who morally disapproved of same-sex sexual behavior, 714 individuals (39 women; 675 men)—corresponding to a demographically weighted overall proportion of 10.5%—expressed moral concerns over same-sex sexual behavior that were consistently stronger than those directed against all other examined sexual matters. Conversely, 5,862 individuals (89.5%) disapproved of one or more of the other examined sexual matters to at least the same extent as they disapproved of same-sex sexual activity. The proportion (10.5%) with particularly strong moral concerns over same-sex sexual activity was higher among men (15.0%) than women (1.7%), a highly statistically significant sex difference (χ^2^ = 288, df = 1, *p* < 0.001). Taken together, our demographically weighted population estimates for the proportion of individuals in Denmark whose moral concerns about sexual matters are particularly pronounced when it comes to same-sex sexual behavior are 0.2% (1.7% × 14.4%) for women and 4.4% (15.0% × 29.2%) for men.

Finally, in a series of supplementary analyses shown in online Tables S2-S5, we repeated all the statistical analyses underlying Tables 2–5, this time utilizing the full dataset by including individuals with unclear moral attitudes toward same-sex sexual activity in the morally concerned group. All main results of the original analyses remained statistically significant in these supplementary analyses, yet aORs were systematically more conservative.

## Discussion

In this nationally representative study, we found that viewing same-sex sexual behavior as immoral, our proxy measure of homophobia, was common in the Danish population, with 14.4% of women and 29.2% of men considering same-sex sexual behavior to be morally unacceptable. Of these morally concerned individuals, however, virtually all women and most men held equal or even stronger moral reservations concerning other sexual matters. Consequently, moral disapproval specifically of homosexuality appears to be extremely uncommon among women (0.2%) and rare among men (4.4%) in Denmark, a country that decriminalized homosexual activity in 1933 and became the first nation in the world to legalize same-sex registered partnerships in 1989 (Graugaard et al., 2004).

To the best of our knowledge, no population-based estimates from other countries exist with which to compare our results, but the general pattern of men holding more punitive views than women concurs with the existing literature (e.g., Herek, 2000; Lingiardi et al., 2016; Santona & Tognasso, 2018). Previous studies have not reached agreement as to whether the gender difference in homophobic attitudes depends on the sex of the targets, that is, whether homophobia is directed specifically toward females or males. While one study showed that men had more negative attitudes than women toward both gay men and lesbians (Santona & Tognasso, 2018), others have suggested that women and men may differ in their attitudes toward gay men, but not in their opinions about lesbians (Lingiardi et al., 2005, 2016). Our findings are in line with both observations, since we observed that only small proportions of both women (0.3%) and men (0.8%) had negative attitudes solely toward female same-sex sexual behavior, while substantially more men (10.7%) than women (0.7%) had negative attitudes solely toward male same-sex sexual behavior. However, even larger proportions of both women (13.4%) and men (17.7%) in our study expressed moral disapproval of both female and male same-sex sexual behaviors.

Different theoretical frameworks have been suggested to explain the higher proportion of male than female individuals with homophobic attitudes. Several authors argue that traditional masculinity is closely entwined with heteronormativity, resulting in strong social pressures for boys to adhere with negative attitudes toward homosexuality, which is perceived as a threat to masculine gender norms (e.g., Hooghe & Meeusen, 2012; Kimmel & Mahler, 2003; Kite & Whitley, 1996; Kite et al., 2021; Lingiardi et al., 2016). Such mechanisms may well be at play in our study, considering the observed larger proportion of individuals with generalized moral disapproval of same-sex sexual activity among men than women as well as the almost exclusively male phenomenon of having moral reservations that are selectively targeted at male same-sex sexual behavior.

A longitudinal study investigating homophobic attitudes during the transition from late adolescence to early adulthood among 2,815 individuals in Belgium showed that homophobia was most pronounced among those with traditional and conservative views on gender roles. This was the case for both sexes, although women generally expressed lower levels of such sexual stereotyping than men (Hooghe & Meeusen, 2012). Furthermore, personality traits such as being rule-bound and conforming with social norms and strict moral codices, as well as high levels of social dominance orientation, have been linked with homophobia (e.g., Lingiardi et al., 2016; Whitley & Ægisdóttir, 2000).

We did not measure personality traits among our participants, but both female and male respondents with moral reservations concerning same-sex sexual behavior had consistently more conservative opinions than those without such reservations regarding other sexual matters such as premarital or casual sex, multiple sex partners, and porn watching. Also, we observed that individuals with moral concerns over same-sex sexual activity were older at sexual debut and reported fewer sex partners, possibly reflecting somewhat restrictive values with respect to their own sexual behaviors. Additionally, we observed that 89.5% of individuals with moralizing views concerning same-sex sexual activity expressed similar or even stronger moral reservations concerning one or more of the other sexual matters examined in our study. These findings align well with the idea that homophobia might reflect restrictiveness toward sexuality in general and not specifically toward homosexuality (Cheval et al., 2016).

We also observed positive associations between moral disapproval of same-sex sexual behavior and a range of other restrictive sexual attitudes. Unexpectedly, however, women with moral concerns over same-sex sexual activity were more tolerant toward infidelity than women without such moral concerns. We have found no prior reports on this association in the literature. Potentially, it might reflect forms of traditional gender norms, in which women may outwardly condemn infidelity as immoral, while tacitly accepting the notion that “men will be men” and that women should stand by their husbands and not expect them to be perfectly faithful.

Women and men with moral concerns over same-sex sexual activity in our study were older, less educated, and reported more financial difficulties than individuals without such concerns. These observations are well in line with those of prior studies (e.g., Obeid et al., 2020; Hooghe & Meeusen, 2012; Lingiardi et al., 2016; Chaux & Léon, 2016). Short education has been found to be positively correlated with homophobic attitudes (Lingiardi et al., 2016; Obeid et al., 2020), and a study comparing homophobic attitudes among adolescents in six Latin American countries has suggested that educational interventions aimed to promote empathy and reduce aggressive attitudes and beliefs could reduce homophobia in adolescents (Chaux & Léon, 2016). Accordingly, in a small study of 128 university students in the USA, the authors observed a significant decrease in homophobia levels among students attending a human sexuality class over the course of a semester (Rogers et al., 2009). Our finding concerning the possible impact of school-based sex education is of relevance in this context. We observed that individuals expressing moral disapproval of same-sex sexual activity were significantly less likely to have received sex education in school than persons without such moral concerns. Consequently, ensuring access to and participation in such education, which has been mandatory in Danish elementary schools since 1970 (Graugaard et al., 2004; Roien et al., 2022), should be considered a potential strategy for reducing the prevalence of homophobic attitudes.

In concordance with previous studies, we observed that individuals who had moral concerns over same-sex sexual activity were markedly more likely than those without such concerns to report that religion was, or had previously been, an important part of their lives (e.g., Hooghe & Meeusen, 2012; Chaux & Léon, 2016; Whitley, 2009; Herek, 1988; Johnson et al., 1997). Results from the abovementioned Belgian study showed marked differences in homophobia across religious groups, with Muslim respondents displaying particularly high levels of homophobic attitudes (Hooghe & Meeusen, 2012). Accordingly, we observed that Muslims in Denmark, but also people belonging to other religious communities such as Jehovah’s Witnesses, Danish Inner Mission, and certain minor Christian denominations, were particularly likely to express moral reservations concerning same-sex sexual activity.

Possible explanations for religious persons tending to hold more negative attitudes toward homosexuality than non-religious persons were discussed in a meta-analysis of 61 studies from North America (Whitley, 2009). One obvious suggestion was that most religious doctrines condemn homosexuality and that religious individuals may internalize and conform with such teachings. Compatible with this idea, we observed that religions with strong anti-homosexual doctrines, including Islam, Jehovah’s Witnesses, Danish Inner Mission, and those branches of Christianity that we analyzed collectively as minor Christian denominations (i.e., Orthodox Christianity, Danish Lutheran Mission, The Pentecostal Movement, and Seventh-day Adventism), displayed the strongest associations with moral disapproval of same-sex sexual activity. However, non-doctrinal factors associated with religiousness (e.g., beliefs regarding the nature and origins of homosexuality) may also play a role. Hence, the stronger people believe that same-sex sexual attraction is controllable and a matter of individual choice, the more negative attitudes they will typically hold against same-sex oriented persons (Whitley, 2009).

Other suggested explanations for the strong link between religiousness and homophobic attitudes include the perception of homosexuals as a threat to traditional family values and/or adherence to right-wing authoritarianism (Whitley, 2009). Although we did not have information about our respondents’ political values or ideological beliefs, we did observe that participants with moral reservations concerning same-sex sexual activity had more restrictive and conservative attitudes toward sexual matters in general. However, in a robustness analysis, in which we reanalyzed the links between moral disapproval of same-sex sexual behavior and attitudes toward other matters relating to sexuality and sexual rights among non-religious individuals only, we observed similar associations. This indicates that moral concerns over same-sex sexual activity and restrictive views on other sexual matters are closely linked, regardless of whether people are religious or not.

Overall, our findings indicate that individuals who find same-sex sexual activity morally unacceptable tend to hold more conservative views and engage in more restrictive sexual behaviors, even among non-religious individuals. These results suggest that moral judgments concerning same-sex sexual behavior are only partially rooted in negative attitudes toward homosexuality per se, and that such sexual prejudice is embedded in a broader network of conservative, authoritarian, and social dominance-oriented belief systems.

### Strengths and Limitations

Our findings were based on a large and nationally representative study covering the age span 15–89 years, and we assessed a broad range of sociodemographic, health-related, sexual, moral, and religious characteristics among individuals with and without moral concerns over same-sex sexual activity. To the best of our knowledge, no prior investigation has accomplished this. Our findings may likely apply to other affluent welfare states and cultures with similar views on sexuality and religion as those prevailing in Denmark. However, what characterizes people with moral reservations concerning same-sex sexual behavior in less sexually accepting countries and cultures may well differ considerably from what we have reported here.

Other limitations also need attention. One is the cross-sectional nature of our self-report questionnaire data. Consequently, while our large study permitted us to provide an unprecedentedly detailed account of multiple factors associated with moral disapproval of same-sex sexual behavior, any causal inferences should be made only with clear reservations. Also, it should be noted that we combined two sex-specific questionnaire items that were created specifically for the Project SEXUS cohort study to measure moral disapproval of same-sex sexual activity in Denmark. All questionnaire items, including those used to capture moral disapproval of same-sex sexual behavior, were cognitively validated during the pilot testing phase, so we have reason to believe that our study provides reliable prevalence estimates of moralizing views against same-sex sexual behavior, the proxy measure of homophobia that we used. However, our epidemiological findings should not be compared uncritically with homophobia prevalence estimates obtained in studies employing more elaborate scales to capture the complex psychosocial construct of homophobia. Acknowledging the methodological trade-offs inherent in any research discipline, we suggest that future research could build on our findings to explore the extent to which interconnected networks of moral concerns about various sexual behaviors are associated with underlying attitude constructs and prejudices.

The way we operationally defined moral disapproval of same-sex sexual activity in our study also deserves attention. In the main analyses, for clarity, we chose to present findings for individuals with or without moral concerns over gay and lesbian sex, leaving out results for individuals with unclear attitudes toward same-sex sexual behavior. However, because previous research has found that ambivalence toward homosexuals is associated with more negative than positive attitudes and with less support of homosexual rights (Hoffarth & Hodson, 2014), another plausible approach would be to include individuals with unclear attitudes in the group with moral reservations about same-sex sexual activity. Consequently, in supplementary analyses, we repeated all statistical analyses with individuals expressing unclear attitudes included in the group who morally disapproved of same-sex sexual behavior. All reported main findings remained statistically significant, although associations in these supplementary analyses were consistently more conservative.

### Conclusion

In several regards, Denmark has been an international frontrunner when it comes to sexual minority rights. However, with an estimated 21.7% of 15–89-year-old Danes deeming same-sex sexual behavior morally unacceptable, it seems clear that such moralizing attitudes remain an important public health concern in this country. Based on our findings, we suggest initiatives aimed at reducing sexual prejudice, such as improving sex education in schools and promoting social inclusion and destigmatization of sexual minorities. Also, religious communities should be encouraged to actively counter homophobia within their circles and beyond. On a broader societal level, continuous attempts should be made to challenge negative attitudes toward sexual minorities, ensuring the rights, health, and well-being of all citizens.

## Supplementary Information

Below is the link to the electronic supplementary material.

Supplementary file1 (DOCX 52 KB)

## Data Availability

The dataset used in this study belongs to Project SEXUS and cannot be shared publicly because of personal health information at the individual level.
