# Supplementary material for: Factors Associated with Moral Disapproval of Same-Sex Sexual Behavior in Denmark: Baseline Findings from the Project SEXUS Cohort Study
Source: Arch Sex Behav. 2025 Jul 9;54(7):2611–23. doi: 10.1007/s10508-025-03211-5 (PMC12457480; doi:10.1007/s10508-025-03211-5)
Supplement: Supplementary file 1 — Supplementary file1 (DOCX 52 KB) [file 10508_2025_3211_MOESM1_ESM.docx]

| **Table S2. Supplementary analysis of sociodemographic and health-related characteristics among 15–89-year-old individuals who did or did not express moral disapproval of same-sex sexual behavior (with individuals expressing unclear attitudes included in the group expressing moral disapproval), Project SEXUS, Denmark 2017-2018** | | | | | | | |
| --- | --- | --- | --- | --- | --- | --- | --- |
|  | **Women** | | |  | **Men** | | |
|  | Moral disapproval of same-sex sexual behavior | | OR [95% CI] |  | Moral disapproval of same-sex sexual behavior | | OR [95% CI] |
|  | No | Yes |  |  | No | Yes |  |
|  | *n* (%) | *n* (%) |  |  | *n* (%) | *n* (%) |  |
| **Total** | 12,326 (68.0) | 4691 (32.0) |  |  | 7461 (52.1) | 7330 (47.9) |  |
| **Age** |  |  |  |  |  |  |  |
| 15-24 years | 2390 (84.5) | 404 (15.5) | 0.51 (0.45-0.59) |  | 1135 (68.6) | 507 (31.4) | 0.49 (0.43-0.56) |
| 25-34 years | 2279 (83.3) | 423 (16.7) | 0.56 (0.49-0.64) |  | 1281 (71.7) | 486 (28.3) | 0.42 (0.37-0.48) |
| 35-44 years | 2307 (81.1) | 511 (18.9) | 0.65 (0.57-0.74) |  | 1459 (65.8) | 736 (34.2) | 0.56 (0.49-0.63) |
| 45-54 years | 2283 (73.7) | 837 (26.3) | 1 [Ref] |  | 1391 (51.6) | 1225 (48.4) | 1 [Ref] |
| 55-64 years | 1822 (66.6) | 919 (33.4) | 1.40 (1.25-1.57) |  | 1203 (44.5) | 1468 (55.5) | 1.33 (1.19-1.49) |
| 65-74 years | 983 (48.6) | 1016 (51.4) | 2.96 (2.62-3.35) |  | 732 (29.9) | 1656 (70.1) | 2.50 (2.22-2.82) |
| ≥ 75 years | 262 (28.9) | 581 (71.1) | 6.88 (5.78-8.19) |  | 260 (16.8) | 1252 (83.2) | 5.31 (4.53-6.21) |
| Median | 44 years | 63 years | p<0.001 |  | 40 years | 56 years | p<0.001 |
| **Region of residence** |  |  |  |  |  |  |  |
| Capital Region of Denmark | 4140 (75.4) | 1055 (24.6) | 1 [Ref] |  | 2766 (65.1) | 1507 (34.9) | 1 [Ref] |
| Region Zealand | 1622 (64.0) | 776 (36.0) | 1.69 (1.49-1.92) |  | 923 (47.6) | 1112 (52.4) | 1.92 (1.69-2.17) |
| Region of Southern Denmark | 2423 (62.5) | 1166 (37.5) | 1.82 (1.63-2.03) |  | 1362 (44.0) | 1840 (56.0) | 2.34 (2.11-2.61) |
| Central Denmark Region | 2762 (68.7) | 1014 (31.3) | 1.46 (1.30-1.63) |  | 1692 (49.9) | 1832 (50.1) | 1.91 (1.73-2.12) |
| North Denmark Region | 1379 (62.3) | 680 (37.7) | 1.87 (1.64-2.14) |  | 718 (43.3) | 1039 (56.7) | 2.38 (2.10-2.69) |
| **Partner status** |  |  |  |  |  |  |  |
| Spouse/Partner | 9024 (68.1) | 3517 (31.9) | 1 [Ref] |  | 5776 (50.6) | 5909 (49.4) | 1 [Ref] |
| Single | 3233 (67.8) | 1146 (32.2) | 0.84 (0.77-0.93) |  | 1644 (56.5) | 1384 (43.5) | 1.15 (1.04-1.27) |
| **Educational attainment** |  |  |  |  |  |  |  |
| ≤ 10 years | 1354 (53.9) | 937 (46.1) | 2.64 (2.32-3.01) |  | 900 (39.9) | 1660 (60.1) | 1.85 (1.65-2.08) |
| Secondary education | 1446 (82.1) | 257 (17.9) | 1.39 (1.17-1.65) |  | 884 (71.0) | 401 (29.0) | 0.77 (0.66-0.89) |
| Short-cycle higher education | 2197 (59.8) | 1294 (40.2) | 1.71 (1.55-1.89) |  | 1029 (49.0) | 1093 (51.0) | 1.10 (0.99-1.23) |
| Medium-cycle higher education | 5130 (69.5) | 1844 (30.5) | 1 [Ref] |  | 2834 (48.3) | 3139 (51.7) | 1 [Ref] |
| Long-cycle higher education | 2120 (83.2) | 311 (16.8) | 0.50 (0.43-0.58) |  | 1768 (66.5) | 932 (33.5) | 0.41 (0.36-0.45) |
| **Difficulties paying bills within the last year** |  |  |  |  |  |  |  |
| Not at all | 10,034 (67.0) | 3959 (33.0) | 1 [Ref] |  | 6425 (51.4) | 6475 (48.6) | 1 [Ref] |
| Sometimes | 1828 (72.5) | 594 (27.5) | 1.25 (1.12-1.40) |  | 881 (57.0) | 712 (43.0) | 1.09 (0.97-1.22) |
| Often | 290 (74.8) | 89 (25.2) | 1.25 (0.96-1.63) |  | 117 (54.3) | 102 (45.7) | 1.36 (1.02-1.82) |
| **Employment status**^a^ |  |  |  |  |  |  |  |
| Working | 8445 (78.1) | 2307 (21.9) | 1 [Ref] |  | 5284 (59.2) | 3683 (40.8) | 1 [Ref] |
| Unemployed | 2525 (75.9) | 758 (24.1) | 1.27 (1.15-1.41) |  | 1133 (63.7) | 695 (36.3) | 0.99 (0.87-1.11) |
| **Self-rated health** |  |  |  |  |  |  |  |
| Very good or good | 9921 (69.5) | 3535 (30.5) | 1 [Ref] |  | 6051 (53.5) | 5625 (46.5) | 1 [Ref] |
| Neither good nor bad | 1735 (63.1) | 815 (36.9) | 1.23 (1.10-1.36) |  | 1050 (47.7) | 1263 (52.3) | 1.17 (1.06-1.29) |
| Bad or very bad | 670 (61.1) | 341 (38.9) | 1.35 (1.16-1.57) |  | 360 (45.6) | 442 (54.4) | 1.16 (0.99-1.36) |
| *OR* Odds ratio adjusted for age in 10-year intervals, *CI* Confidence interval, *n* (%) Numbers of respondents with characteristic and corresponding demographically weighted row percentages  ^a^Analysis restricted to persons under the age of 65 years | | | | | | | |

| **Table S3. Supplementary analysis of sexual characteristics among 15–89-year-old individuals who did or did not express moral disapproval of same-sex sexual behavior (with individuals expressing unclear attitudes included in the group expressing moral disapproval), Project SEXUS, Denmark 2017-2018** | | | | | | | |
| --- | --- | --- | --- | --- | --- | --- | --- |
|  | **Women** | | |  | **Men** | | |
|  | Moral disapproval of same-sex sexual behavior | | aOR [95% CI] |  | Moral disapproval of same-sex sexual behavior | | aOR [95% CI] |
|  | No | Yes |  |  | No | Yes |  |
|  | *n* (%) | *n* (%) |  |  | *n* (%) | *n* (%) |  |
| **Overall sexual experience** |  |  |  |  |  |  |  |
| None | 585 (66.1) | 258 (33.9) | 2.94 (2.34-3.70) |  | 376 (60.6) | 263 (39.4) | 1.20 (0.97-1.49) |
| Any | 11,709 (68.6) | 4329 (31.4) | 1 [Ref] |  | 7072 (51.7) | 7017 (48.3) | 1 [Ref] |
| **Sexual identity^a^** |  |  |  |  |  |  |  |
| Heterosexual | 11,152 (69.5) | 4130 (30.5) | 1 [Ref] |  | 6685 (51.8) | 6872 (48.2) | 1 [Ref] |
| Homosexual | 302 (93.4) | 15 (6.6) | 0.22 (0.10-0.45) |  | 473 (95.6) | 24 (4.4) | 0.06 (0.03-0.12) |
| Bisexual | 463 (89.9) | 45 (10.1) | 0.38 (0.27-0.54) |  | 196 (81.7) | 45 (18.3) | 0.20 (0.14-0.29) |
| **Other-sex sexual experience** |  |  |  |  |  |  |  |
| None | 672 (66.7) | 269 (33.3) | 2.69 (2.16-3.35) |  | 627 (65.4) | 276 (34.6) | 0.87 (0.71-1.06) |
| Any | 11,622 (68.6) | 4318 (31.4) | 1 [Ref] |  | 6821 (51.3) | 7004 (48.7) | 1 [Ref] |
| **Same-sex sexual experience** |  |  |  |  |  |  |  |
| None | 10,720 (66.7) | 4406 (33.3) | 1 [Ref] |  | 6488 (50.3) | 7118 (49.7) | 1 [Ref] |
| Any | 1574 (87.5) | 181 (12.5) | 0.39 (0.33-0.47) |  | 960 (82.9) | 162 (17.1) | 0.21 (0.17-0.26) |
| **Same-sex sexual attraction** |  |  |  |  |  |  |  |
| None | 7730 (61.6) | 4044 (38.4) | 1 [Ref] |  | 5804 (47.9) | 6932 (52.1) | 1 [Ref] |
| Any | 4508 (87.5) | 539 (12.5) | 0.37 (0.33-0.41) |  | 1615 (83.1) | 310 (16.9) | 0.23 (0.20-0.26) |
| **Age at sexual debut with a person of the other sex^b^** |  |  |  |  |  |  |  |
| < 12 years | 73 (69.5) | 28 (30.5) | 1.10 (0.68-1.79) |  | 48 (42.3) | 61 (57.7) | 1.54 (0.95-2.51) |
| 12-14 years | 1995 (81.4) | 428 (18.6) | 0.80 (0.70-0.91) |  | 917 (57.8) | 702 (42.2) | 1.01 (0.89-1.15) |
| 15-17 years | 6544 (71.5) | 2242 (28.5) | 1 [Ref] |  | 3297 (54.1) | 3056 (45.9) | 1 [Ref] |
| 18-20 years | 2354 (60.8) | 1152 (39.2) | 1.26 (1.13-1.40) |  | 1785 (47.1) | 2134 (52.9) | 1.05 (0.95-1.15) |
| > 20 years | 579 (48.8) | 439 (51.2) | 2.22 (1.86-2.65) |  | 738 (43.2) | 1017 (56.8) | 1.24 (1.09-1.42) |
| **Total number of partners of the other sex^b^** |  |  |  |  |  |  |  |
| 1 | 973 (49.7) | 777 (50.3) | 3.06 (2.62-3.57) |  | 589 (43.3) | 893 (56.7) | 1.81 (1.56-2.09) |
| 2 | 970 (55.1) | 567 (44.9) | 2.51 (2.14-2.94) |  | 477 (43.2) | 645 (56.8) | 1.77 (1.50-2.07) |
| 3-4 | 1911 (60.3) | 995 (39.7) | 2.12 (1.87-2.41) |  | 1047 (46.3) | 1336 (53.7) | 1.41 (1.26-1.58) |
| 5-9 | 2988 (72.7) | 965 (27.3) | 1.45 (1.29-1.64) |  | 1654 (52.8) | 1582 (47.2) | 1.14 (1.03-1.26) |
| ≥ 10 | 4413 (82.7) | 834 (17.3) | 1 [Ref] |  | 2907 (57.7) | 2257 (42.3) | 1 [Ref] |
| **Rating of current sex life** |  |  |  |  |  |  |  |
| Very good or good | 6215 (72.8) | 2035 (27.2) | 1 [Ref] |  | 3881 (56.3) | 3186 (43.7) | 1 [Ref] |
| Neither good nor bad | 2734 (69.7) | 1002 (30.3) | 1.00 (0.90-1.12) |  | 1871 (51.4) | 1870 (48.6) | 1.13 (1.02-1.24) |
| Bad or very bad | 1471 (74.2) | 438 (25.8) | 0.93 (0.81-1.06) |  | 1120 (51.8) | 1126 (48.2) | 1.08 (0.96-1.21) |
| No sex life within the last year | 1807 (54.6) | 1123 (45.4) | 1.22 (1.07-1.39) |  | 550 (37.1) | 1097 (62.9) | 1.53 (1.31-1.78) |
| **Received sex education in school** |  |  |  |  |  |  |  |
| Yes | 10,711 (75.2) | 3200 (24.8) | 1 [Ref] |  | 6313 (58.9) | 4560 (41.1) | 1 [Ref] |
| No | 1129 (41.8) | 1195 (58.2) | 1.46 (1.29-1.66) |  | 917 (28.2) | 2467 (71.8) | 1.53 (1.36-1.71) |
| **Communication with parents about sex** |  |  |  |  |  |  |  |
| To a very high or high extent | 1283 (78.7) | 310 (21.3) | 0.92 (0.78-1.08) |  | 373 (70.4) | 162 (29.6) | 0.78 (0.61-0.99) |
| To some extent | 2906 (75.3) | 842 (24.7) | 1 [Ref] |  | 1195 (63.7) | 701 (36.3) | 1 [Ref] |
| To a low extent | 4217 (69.5) | 1498 (30.5) | 0.99 (0.89-1.11) |  | 2692 (57.7) | 2097 (42.3) | 1.03 (0.91-1.17) |
| Not at all | 3830 (60.3) | 1970 (39.7) | 1.18 (1.05-1.32) |  | 3144 (43.6) | 4295 (56.4) | 1.37 (1.21-1.55) |
| *aOR* Odds ratio adjusted for age in 10-year intervals, region of residence, partner status, educational attainment, difficulties paying bills within the last year and self-rated health, *CI* Confidence interval, *n* (%) Numbers of respondents with characteristic and corresponding demographically weighted row percentages  ^a^Sexual identity categories *Asexual*, *I cannot be placed in the abovementioned categories*, and *I do not know/undecided* were not included in the analyses  ^b^Among individuals with heterosexual experience | | | | | | | |

| **Table S4. Supplementary analysis of moral attitudes towards other matters related to sexuality and sexual rights among 15–89-year-old individuals who did or did not express moral disapproval of same-sex sexual behavior (with individuals expressing unclear attitudes included in the group expressing moral disapproval), Project SEXUS, Denmark 2017-2018** | | | | | | | |
| --- | --- | --- | --- | --- | --- | --- | --- |
|  | **Women** | | |  | **Men** | | |
|  | Moral disapproval of same-sex sexual behavior | | aOR [95% CI] |  | Moral disapproval of same-sex sexual behavior | | aOR [95% CI] |
|  | No | Yes |  |  | No | Yes |  |
|  | *n* (%) | *n* (%) |  |  | *n* (%) | *n* (%) |  |
| ***To what extent do you consider it morally acceptable…*** | | |  |  |  |  |  |
| **That some people have sex without being married to each other?** |  |  |  |  |  |  |  |
| To a very high or high extent | 12,001 (78.8) | 2766 (21.2) | 1 [Ref] |  | 7227 (60.8) | 4921 (39.2) | 1 [Ref] |
| To some extent | 149 (17.2) | 619 (82.8) | 12.52 (9.86-15.88) |  | 105 (11.2) | 850 (88.8) | 8.50 (6.65-10.85) |
| To a low extent or not at all | 145 (12.3) | 931 (87.7) | 20.71 (16.36-26.23) |  | 108 (7.7) | 1242 (92.3) | 14.89 (11.78-18.84) |
| **That some people have sex with a person they just met?** |  |  |  |  |  |  |  |
| To a very high or high extent | 8082 (90.9) | 729 (9.1) | 1 [Ref] |  | 6131 (70.7) | 2613 (29.3) | 1 [Ref] |
| To some extent | 2865 (63.8) | 1468 (36.2) | 4.37 (3.91-4.88) |  | 982 (31.8) | 2295 (68.2) | 4.08 (3.70-4.51) |
| To a low extent or not at all | 1099 (31.6) | 1984 (68.4) | 13.80 (12.19-15.63) |  | 287 (13.3) | 2017 (86.7) | 11.27 (9.67-13.13) |
| **That some people have many changing sex partners?** |  |  |  |  |  |  |  |
| To a very high or high extent | 6792 (94.7) | 342 (5.3) | 1 [Ref] |  | 5384 (77.8) | 1561 (22.2) | 1 [Ref] |
| To some extent | 3132 (71.5) | 1131 (28.5) | 5.78 (5.02-6.66) |  | 1375 (39.4) | 2282 (60.6) | 4.54 (4.12-5.01) |
| To a low extent or not at all | 2005 (38.5) | 2686 (61.5) | 18.31 (15.92-21.05) |  | 595 (17.3) | 3040 (82.7) | 12.05 (10.68-13.59) |
| **That some people have sex with more than one person at the same time?** |  |  |  |  |  |  |  |
| To a very high or high extent | 7013 (96.8) | 206 (3.2) | 1 [Ref] |  | 5872 (80.3) | 1435 (19.7) | 1 [Ref] |
| To some extent | 2600 (72.9) | 929 (27.1) | 10.06 (8.46-11.95) |  | 994 (34.8) | 1956 (65.2) | 6.73 (6.05-7.48) |
| To a low extent or not at all | 2027 (37.6) | 2898 (62.4) | 33.57 (28.25-39.90) |  | 454 (12.5) | 3311 (87.5) | 20.78 (18.17-23.76) |
| **That some people watch porn?** |  |  |  |  |  |  |  |
| To a very high or high extent | 9993 (92.1) | 804 (7.9) | 1 [Ref] |  | 7151 (69.0) | 3285 (31.0) | 1 [Ref] |
| To some extent | 1653 (46.4) | 1668 (53.6) | 11.25 (10.05-12.58) |  | 213 (9.5) | 2090 (90.5) | 16.66 (14.21-19.54) |
| To a low extent or not at all | 499 (21.0) | 1566 (79.0) | 33.10 (28.65-38.23) |  | 82 (5.1) | 1543 (94.9) | 31.00 (24.06-39.95) |
| **That some people pay other persons for sex?** |  |  |  |  |  |  |  |
| To a very high or high extent | 2365 (88.3) | 287 (11.7) | 0.18 (0.16-0.21) |  | 3199 (70.8) | 1371 (29.2) | 0.26 (0.23-0.28) |
| To some extent | 3179 (72.7) | 1006 (27.3) | 0.60 (0.54-0.66) |  | 2318 (51.7) | 2330 (48.3) | 0.58 (0.53-0.64) |
| To a low extent or not at all | 6320 (64.2) | 2798 (35.8) | 1 [Ref] |  | 1805 (38.4) | 3134 (61.6) | 1 [Ref] |
| **That some people receive payment for sex?** |  |  |  |  |  |  |  |
| To a very high or high extent | 2585 (89.3) | 277 (10.7) | 0.16 (0.14-0.19) |  | 3518 (73.4) | 1328 (26.6) | 0.19 (0.17-0.21) |
| To some extent | 3524 (75.9) | 938 (24.1) | 0.47 (0.43-0.52) |  | 2286 (51.1) | 2356 (48.9) | 0.51 (0.46-0.56) |
| To a low extent or not at all | 5706 (61.3) | 2879 (38.7) | 1 [Ref] |  | 1517 (34.3) | 3130 (65.7) | 1 [Ref] |
| **That some people are unfaithful to their partner?** |  |  |  |  |  |  |  |
| To a very high or high extent | 193 (50.3) | 182 (49.7) | 1.45 (1.08-1.94) |  | 362 (48.0) | 374 (52.0) | 0.78 (0.64-0.94) |
| To some extent | 1258 (75.8) | 332 (24.2) | 0.58 (0.50-0.68) |  | 1372 (55.4) | 1088 (44.6) | 0.72 (0.64-0.80) |
| To a low extent or not at all | 10,589 (68.9) | 3867 (31.1) | 1 [Ref] |  | 5520 (52.5) | 5509 (47.5) | 1 [Ref] |
| ***Do you believe that, in Denmark, it should remain legal…*** | |  | |  |  |  | |
| **For two persons of the same sex to marry each other?** |  |  |  |  |  |  |  |
| Yes | 12,230 (73.5) | 3661 (26.5) | 1 [Ref] |  | 7290 (60.2) | 5111 (39.8) | 1 [Ref] |
| No | 55 (9.2) | 559 (90.8) | 25.15 (17.25-36.67) |  | 103 (6.7) | 1475 (93.3) | 16.39 (12.95-20.75) |
| **For parents to have their sons under 18 years of age circumcised without a medical indication?** |  |  |  |  |  |  |  |
| Yes | 529 (64.4) | 247 (35.6) | 2.14 (1.72-2.67) |  | 426 (55.5) | 334 (44.5) | 1.47 (1.22-1.78) |
| No | 11,009 (68.0) | 4180 (32.0) | 1 [Ref] |  | 6514 (51.3) | 6628 (48.7) | 1 [Ref] |
| **To get an abortion due to unwanted pregnancy (before the end of 12^th^ week of pregnancy)?** |  |  |  |  |  |  |  |
| Yes | 11,889 (71.8) | 3800 (28.2) | 1 [Ref] |  | 7131 (55.4) | 6147 (44.6) | 1 [Ref] |
| No | 197 (25.0) | 488 (75.0) | 8.36 (6.74-10.38) |  | 125 (17.7) | 611 (82.3) | 5.60 (4.36-7.20) |
| *aOR* Odds ratio adjusted for age in 10-year intervals, region of residence, partner status, educational attainment, difficulties paying bills within the last year and self-rated health, *CI* Confidence interval, *n* (%) Numbers of respondents with characteristic and corresponding demographically weighted row percentages | | | | | | | |

| **Table S5. Supplementary analysis of religious characteristics among 15–89-year-old individuals who did or did not express moral disapproval of same-sex sexual behavior (with individuals expressing unclear attitudes included in the group expressing moral disapproval), Project SEXUS, Denmark 2017-2018** | | | | | | | |
| --- | --- | --- | --- | --- | --- | --- | --- |
|  | **Women** | | |  | **Men** | | |
|  | Moral disapproval of same-sex sexual behavior | | aOR [95% CI] |  | Moral disapproval of same-sex sexual behavior | | aOR [95% CI] |
|  | No | Yes |  |  | No | Yes |  |
|  | *n* (%) | *n* (%) |  |  | *n* (%) | *n* (%) |  |
| **Importance of religion in childhood home** |  |  |  |  |  |  |  |
| Not at all important | 4578 (79.0) | 1065 (21.0) | 1 [Ref] |  | 3131 (64.1) | 1833 (35.9) | 1 [Ref] |
| Not very important | 5504 (70.7) | 1895 (29.3) | 1.23 (1.12-1.36) |  | 3236 (51.4) | 3251 (48.6) | 1.29 (1.18-1.41) |
| Somewhat important | 1697 (54.2) | 1060 (45.8) | 1.83 (1.62-2.07) |  | 798 (36.2) | 1491 (63.8) | 1.93 (1.70-2.19) |
| Very important | 446 (41.5) | 575 (58.5) | 4.35 (3.60-5.26) |  | 255 (27.2) | 650 (72.8) | 3.89 (3.18-4.76) |
| **Specific religion in childhood home^a^** |  |  |  |  |  |  |  |
| Not at all important | 4578 (79.0) | 1065 (21.0) | 1 [Ref] |  | 3131 (64.1) | 1833 (35.9) | 1 [Ref] |
| Lutheran-evangelical Christianity (Danish State Church) | 6405 (65.8) | 2577 (34.2) | 1.19 (1.08-1.31) |  | 3698 (47.6) | 4348 (52.4) | 1.30 (1.19-1.42) |
| Catholicism | 144 (60.1) | 81 (39.9) | 2.70 (1.93-3.78) |  | 80 (44.0) | 119 (56.0) | 2.13 (1.51-3.01) |
| Danish Inner Mission | 74 (45.4) | 77 (54.6) | 1.67 (1.10-2.53) |  | 35 (21.6) | 114 (78.4) | 2.71 (1.68-4.40) |
| Independent Christian constituency | 423 (60.7) | 243 (39.3) | 2.13 (1.74-2.60) |  | 132 (37.3) | 240 (62.7) | 2.51 (1.95-3.25) |
| Jehovah’s Witnesses | 28 (36.1) | 49 (63.9) | 4.58 (2.60-8.07) |  | 16 (19.1) | 62 (80.9) | 6.93 (3.48-13.81) |
| Islam | 81 (31.6) | 186 (68.4) | 21.24 (15.39-29.30) |  | 33 (20.0) | 138 (80.0) | 15.51 (10.04-23.96) |
| Minor Christian denomination^b^ | 39 (46.7) | 48 (53.3) | 4.59 (2.38-8.83) |  | 31 (31.8) | 68 (68.2) | 3.82 (2.19-6.65) |
| Other religion^c^ | 73 (61.3) | 42 (38.7) | 2.18 (1.32-3.60) |  | 49 (50.1) | 56 (49.9) | 1.71 (1.08-2.72) |
| **Importance of religion today** |  |  |  |  |  |  |  |
| Not at all important | 4193 (80.8) | 858 (19.2) | 1 [Ref] |  | 3723 (66.0) | 2009 (34.0) | 1 [Ref] |
| Not very important | 5192 (70.6) | 1812 (29.4) | 1.36 (1.23-1.51) |  | 2675 (47.3) | 3172 (52.7) | 1.65 (1.51-1.80) |
| Somewhat important | 2450 (59.2) | 1313 (40.8) | 1.62 (1.44-1.83) |  | 856 (36.1) | 1562 (63.9) | 2.22 (1.97-2.50) |
| Very important | 351 (33.6) | 609 (66.4) | 6.08 (4.95-7.46) |  | 157 (20.7) | 520 (79.3) | 6.31 (4.91-8.09) |
| **Specific religion today^a^** |  |  |  |  |  |  |  |
| Not at all important | 4193 (80.8) | 858 (19.2) | 1 [Ref] |  | 3723 (66.0) | 2009 (34.0) | 1 [Ref] |
| Lutheran-evangelical Christianity (Danish State Church) | 6589 (65.0) | 2759 (35.0) | 1.28 (1.15-1.41) |  | 3046 (42.6) | 4346 (57.4) | 1.70 (1.56-1.86) |
| Catholicism | 95 (52.1) | 71 (47.9) | 4.22 (2.91-6.13) |  | 59 (45.8) | 80 (54.2) | 2.54 (1.65-3.91) |
| Danish Inner Mission | 10 (16.2) | 40 (83.8) | 6.89 (3.17-14.97) |  | 5 (7.2) | 44 (92.8) | 12.68 (3.56-45.12) |
| Independent Christian constituency | 445 (56.8) | 300 (43.2) | 2.72 (2.24-3.29) |  | 119 (34.0) | 233 (66.0) | 3.12 (2.38-4.09) |
| Jehovah’s Witnesses | 3 (8.1) | 43 (91.9) | 37.93 (10.72-134.24) |  | 0 (0) | 52 (100.0) | ∞ |
| Islam | 61 (25.8) | 183 (74.2) | 30.82 (21.66-43.86) |  | 25 (16.7) | 127 (83.3) | 21.26 (13.03-34.67) |
| Minor Christian denomination^b^ | 25 (29.6) | 58 (70.4) | 10.25 (5.15-20.40) |  | 10 (11.9) | 56 (88.1) | 19.09 (8.61-42.32) |
| Other religion^c^ | 234 (76.9) | 67 (23.1) | 0.97 (0.67-1.39) |  | 122 (59.0) | 85 (41.0) | 1.34 (0.96-1.87) |
| *aOR* Odds ratio adjusted for age in 10-year intervals, region of residence, partner status, educational attainment, difficulties paying bills within the last year and self-rated health, *CI* Confidence interval, *n* (%) Numbers of respondents with characteristic and corresponding demographically weighted row percentages  ^a^Analysis restricted to respondents with affiliation to only one religion  ^b^Including Orthodox Christianity, Danish Lutheran Mission, The Pentecostal Movement and Seventh-day Adventism  ^c^Including Judaism, Hinduism, Buddhism, Sikhism, Scientology, and Other religion | | | | | | | |
